# Increased *ompW* and *ompA* expression and higher virulence of *Acinetobacter baumannii* persister cells

**DOI:** 10.1186/s12866-023-02904-y

**Published:** 2023-05-29

**Authors:** Brenda Landvoigt Schmitt, Bruna Ferreira Leal, Mariana Leyser, Muriel Primon de Barros, Danielle Silva Trentin, Carlos Alexandre Sanchez Ferreira, Sílvia Dias de Oliveira

**Affiliations:** 1 Laboratório de Imunologia e Microbiologia, Escola de Ciências da Saúde e da Vida, Pontifícia Universidade Católica do Rio Grande do Sul, PUCRS, Porto Alegre, Brazil; 2 Programa de Pós-Graduação em Biologia Celular e Molecular, Escola de Ciências da Saúde e da Vida, Pontifícia Universidade Católica do Rio Grande do Sul, PUCRS, Porto Alegre, Brazil; 3 Laboratório de Bacteriologia e Modelos Experimentais Alternativos, Departamento de Ciências Básicas da Saúde, Universidade Federal de Ciências da Saúde de Porto Alegre, UFCSPA, R. Sarmento Leite, 245, Porto Alegre, RS 90050-170 Brazil

**Keywords:** *Acinetobacter baumannii*, Outer membrane protein, Persistence, Meropenem, *Galleria mellonella*, Virulence

## Abstract

**Background:**

*Acinetobacter baumannii* is one of the main causes of healthcare-associated infections that threaten public health, and carbapenems, such as meropenem, have been a therapeutic option for these infections. Therapeutic failure is mainly due to the antimicrobial resistance of *A*. *baumannii*, as well as the presence of persister cells. Persisters constitute a fraction of the bacterial population that present a transient phenotype capable of tolerating supra-lethal concentrations of antibiotics. Some proteins have been suggested to be involved in the onset and/or maintenance of this phenotype. Thus, we investigated the mRNA levels of the *adeB* (AdeABC efflux pump component), *ompA*, and *ompW* (outer membrane proteins) in *A*. *baumannii* cells before and after exposure to meropenem.

**Results:**

We found a significant increase (*p-value* < 0.05) in the expression of *ompA* (> 5.5-fold) and *ompW* (> 10.5-fold) in persisters. However, *adeB* did not show significantly different expression levels when comparing treated and untreated cells. Therefore, we suggest that these outer membrane proteins, especially OmpW, could be part of the mechanism of *A*. *baumannii* persisters to deal with the presence of high doses of meropenem. We also observed in the *Galleria mellonella* larvae model that persister cells are more virulent than regular ones, as evidenced by their LD_50_ values.

**Conclusions:**

Taken together, these data contribute to the understanding of the phenotypic features of *A*. *baumannii* persisters and their relation to virulence, as well as highlight OmpW and OmpA as potential targets for drug development against *A*. *baumannii* persisters.

## Background

Healthcare-associated infections (HAIs) are a major cause of morbidity and mortality in patients hospitalized in healthcare institutions and represent a significant proportion of hospitalization costs [1]. *Acinetobacter baumannii* is one of the most prevalent microorganisms in HAIs [2, 3], including cases of ventilator-associated pneumonia, sepsis, and wound infections in patients hospitalized in intensive care units [4]. The main therapeutic choice for these infections is carbapenems, such as meropenem.

Therapeutic failure in infections caused by *A*. *baumannii* is usually related to antibiotic resistance, but it may also be due to the presence of persister cells [5]. Persisters are a small fraction of transient phenotypic variants capable of tolerating supra-lethal concentrations of antimicrobials to which they are genetically susceptible [6]. The clinical impact of persister cells is given by their ability to resume growth when drug levels fall below the therapeutic dose and selective pressure is reduced, allowing the reestablishment of the infection, which can become chronic [7].

The pathways involved in the regulation of persister cell formation have not yet been fully elucidated [8]. However, several molecular mechanisms have been associated with this phenotype, including drug target inactivity, reduced cellular energy, interruption of DNA replication and blocking of translation, intrinsic defenses that act against damage caused by stressors such as antimicrobials, and reduction of the intracellular concentration of these drugs [9]. Thus, membrane proteins, as part of the efflux pump apparatus or not, could play an important role in survival after exposure to antimicrobials. The AdeABC efflux pump is responsible for the extrusion of antibiotics, conferring multidrug resistance in *A*. *baumannii* [10], but when exposed to high doses of tobramycin or colistin, this bacterium showed downregulation of the *adeB* gene [11]. OmpA provides structural rigidity [12, 13] and is implicated in the regulation of outer membrane stability, permeability to small solutes, and antibiotic resistance. In addition, OmpA is able to contribute to the dissemination of *A*. *baumannii* during infection [14], and its overexpression is associated with pneumonia and bacteremia [15]. OmpW forms channels for the uptake of small molecules [16], and the regulation of its expression appears to be essential for bacterial adaptive response to various stress conditions [17, 18]. Given its importance for pathogenesis, both OmpA and OmpW were already suggested as targets for the development of vaccines against *A*. *baumannii* [13, 14, 19–21].

Studies using *Galleria mellonella* larvae have demonstrated that this experimental model can be useful and reliable for analyzing the pathogenicity mechanisms of *A*. *baumannii*, providing a comprehensive comparison of various virulence factors and offering therapeutic strategies for clinical treatments of *A*. *baumannii* infection [22]. For instance, the effect of surface antigen protein 1 was examined during infection of *G. mellonella* with *A*. *baumannii* and the knockout strain presented a lower survival than the wild type, demonstrating the importance of this protein on *A*. *baumannii* virulence [23]. On the other hand, although outer membrane proteins (OMPs) have been identified as virulence factors of *A*. *baumannii* infecting macrophages and murine models [24, 25], they were not investigated in the *G*. *mellonella* host. Likewise, to the best of our knowledge, the virulence of persisters has not yet been studied in *G*. *mellonella*. In this context, we investigated the mRNA expression levels of membrane proteins OmpA, OmpW, and AdeB in both regular and persister cells of *A*. *baumannii* after being exposed to meropenem. We also assessed the virulence of both phenotypes in *G*. *mellonella* larvae.

## Results and discussion

Persister cells were first described in *S*. *aureus* in 1944 [26], and since then, several attempts have been made to understand the pathways and structural differences involved in this transient bacterial phenotype. In this sense, despite the diverse mechanisms being studied, the possible roles performed by membrane proteins in persistence are still relatively poorly investigated. Thus, we evaluated the differential expression of genes encoding outer membrane and efflux pump components in *A*. *baumannii* persister cells after exposure to meropenem. Additionally, we assessed the virulence of these persisters in an animal model for the first time.

**Fig. 1:**
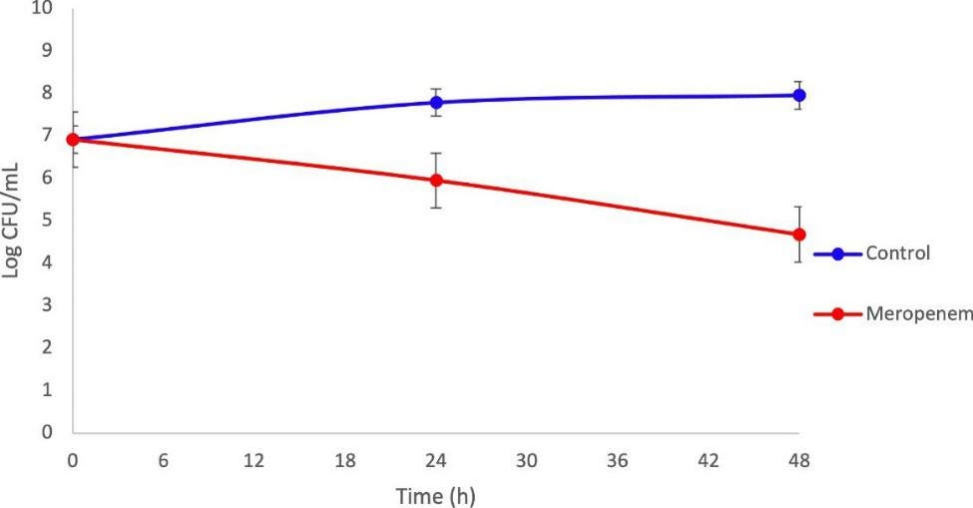
Killing curves of *Acinetobacter baumannii* Acb-1 after 48 h-exposure to meropenem. Acb-1 was cultured until the middle exponential phase and exposed to meropenem at 15 µg/mL, in triplicate, for 48 h. Control (without the addition of the antimicrobial) is represented by a blue line and the treatment with meropenem by a red line

The assay in which meropenem was added to middle logarithm phase Acb-1 cells showed the presence of *bona fide* persister cells after 48 h of exposure at 15 µg/mL (Fig. 1), with levels reaching 0.3181% of the initial population. This result lies in the range of persister fractions already described for this strain [27]. Likewise, when comparing the surviving cell counts of the exposed culture to those of the control culture (not exposed to the antimicrobial) after 48 h of exposure, the surviving fraction was found to be 0.089% (Fig. 1).

**Fig. 2:**
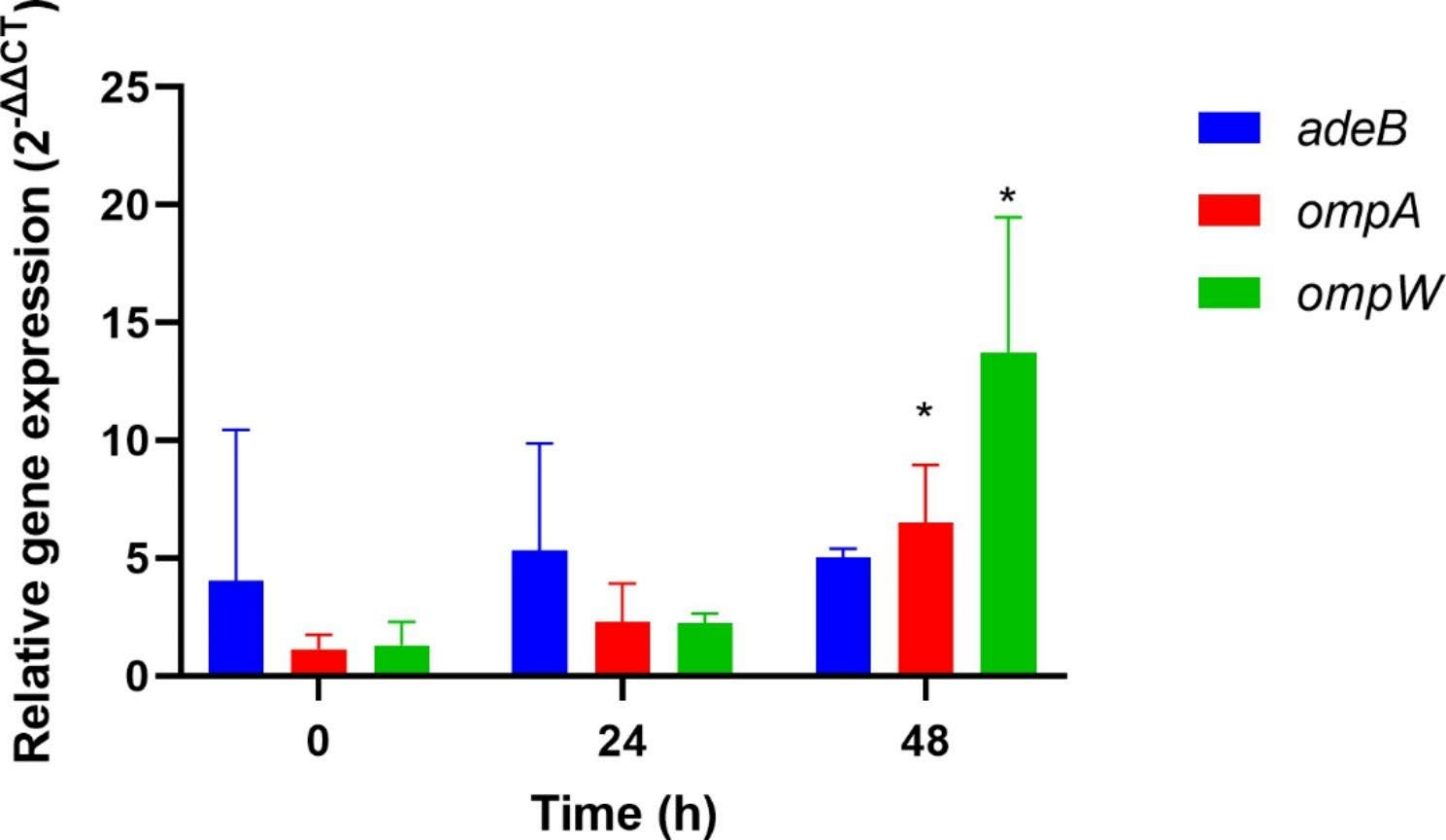
Differential expression of *adeB*, *ompA*, and *ompW* in *Acinetobacter baumannii* regular and persister cells. *rpoB* was used as the control gene in quantitative real-time PCR analysis. Values represent the means of three experimental replicates from three independent experiments. The *y*-axis represents the fold difference of each gene to the threshold cycle (C_T_) values, whereas the *x*-axis indicates the time points of meropenem exposure. Asterisks indicate significant differences between the 0 and 48-h time points after meropenem exposure, as determined by ANOVA followed by Tukey’s multiple comparison test (*p-value* < 0.05)

The differential expression of *adeB*, *ompA*, and *ompW* was evaluated in response to meropenem exposure up to 48 h. We observed that the *ompW* and *ompA* genes were upregulated by more than 10.5-fold and 5.5-fold, respectively, in persisters compared to regular cells (*p-value* < 0.05) (Table 1; Fig. 2).

**Table 1:** Relative expression levels of *adeB*, *omp*A, and *ompW* after meropenem exposure up to 48 h in *Acinetobacter baumannii*

Gene	Differential expression analysis (2^-∆∆Ct^ values)				p-value*
	Before exposure	24 h-exposure		48 h-exposure	
*adeB*	4.02835092	5.33537118		5.04481689	0.8606
*ompA*	1.14363601		2.29038521	6.50678510	0.0157
*ompW*	1.30034341	2.24766680		13.7371198	0.0462

*p-values refer to 48 h-exposure

OmpA is the most abundant porin found in *A*. *baumannii* and plays critical roles in various cellular processes, including antibiotic resistance, host cell invasiveness, biofilm formation, eukaryotic cell infection, and immunomodulation [25, 28–30]. OmpA is known to be involved in the extrusion of antibacterial compounds from the periplasmic region and may be coupled to efflux systems in the inner membrane [30], suggesting that increased *ompA* expression can help *A*. *baumannii* survive exposure to meropenem by increasing drug extrusion. In this sense, mutations in OmpA have been linked to increased susceptibility to meropenem [31]. An increased production of OmpA has also been induced by oxidative stress via different stressors, suggesting that it may play a role in maintaining cellular homeostasis [32]. Another issue to be highlighted is the importance of OmpA in biofilm formation by *A*. *baumannii* [29], which is associated with high levels of persisters [27].

OmpW is a β-barrel protein involved in transporting hydrophobic molecules across the outer membrane, maintaining cellular homeostasis under stress, and seems to be a virulence factor necessary for *A*. *baumannii* pathogenesis without affecting its growth [24]. Interestingly, OmpW is a porin with a pivotal role in the uptake of nutrients, such as iron [33], which may influence the treatment of infections caused by this microorganism [34]. Additionally, deletion of OmpW from *Escherichia coli* resulted in decreased minimum inhibitory concentrations (MICs) of several antibiotics [35], suggesting that the overexpression of *ompW* can be a strategy of *A*. *baumannii* to cope with the exposure to high doses of meropenem. Furthermore, both OmpW and OmpA have been found to bind carbapenemases, which are enzymes capable of hydrolyzing carbapenems and other beta-lactams [31]. Increased amounts of these porins could result in a higher presence of these enzymes in the periplasmic space, leading to a decrease in drug availability to their molecular targets. However, it is noteworthy that OmpW is not a general requirement for persisters, as *A*. *baumannii* exposed to high concentrations of tobramycin showed reduced *ompW* expression [36], indicating that the strategy involving OmpW may be drug dependent. On the other hand, it is also possible that a more generalized response is involved, including protection against the effects of antimicrobials. For instance, *ompW* expression in *Vibrio cholerae* is altered in response to different concentrations of salts, nutrients, oxygen, and changes in temperature [37].

OmpW has been demonstrated to protect *E*. *coli* against host responses by conferring resistance to complement-mediated killing and phagocytosis, suggesting its potential as a drug target in gram-negative bacteria for the development of new therapeutic agents [24]. Furthermore, docking studies and the effects of specific inhibitors have identified both OmpA and OmpW as potential targets for drug development against *A*. *baumannii* infections [13, 38, 39]. Given its important role in transport of essential nutrients, OmpW has also been shown to be a highly immunogenic protein and a candidate for the development of an effective vaccine to control *A*. *baumannii* infections [19]. Thus, our findings extend the potential utility of these proteins as targets for drugs and vaccines against *A*. *baumannii* persisters.

Although the AcrAB-TolC efflux pump has been shown to play a role in persistence by mediating drug efflux in *E*. *coli* [40], *ade*B did not show different expression levels comparing regular and persister cells in our study. Despite being one of the most prevalent efflux pumps in *A*. *baumannii* and conferring multidrug resistance, including against meropenem [41], AdeABC does not appear to be involved in persistence in the face of this antimicrobial agent. However, a significant downregulation of this gene was observed following exposure to tobramycin and colistin in this microorganism [11, 36].

**Fig. 3:**
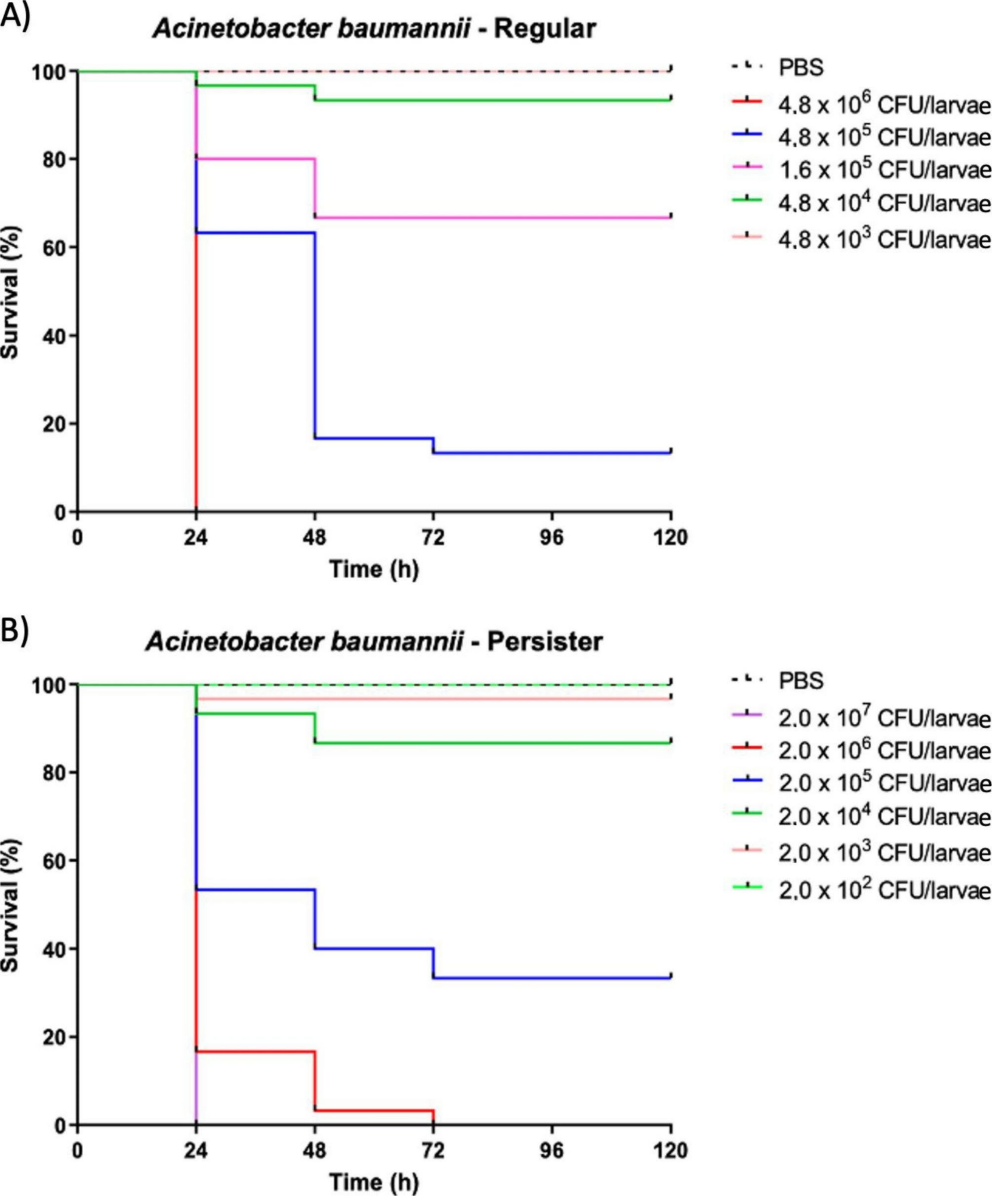
Kaplan-Meier survival curves of *Galleria mellonella* larvae inoculated with *Acinetobacter baumannii* Acb-1 strain: (**A**) regular cells and (**B**) persister cells after exposure to meropenem (15-fold MIC). Vehicle group (phosphate-buffered saline - PBS) was used as sterility and injection control. The data are composed of the mean of three independent experiments with ten larvae in each group

OMPs deal directly with the flux trade of soluble components between the extracellular and intracellular compartments, as well as in the periplasmic space, in gram-negative bacteria. Therefore, they are natural candidates to be modulated when these cells face different environmental conditions, such as starvation or exposure to toxic substances. OmpA and OmpW appear to behave in such a fashion when cells are challenged with meropenem, altering the outer membrane functioning profile, and possibly, also the periplasmic and surrounding extracellular composition. Further research will be important to unravel the molecular mechanisms underlying the contribution of overexpressed OmpW and OmpA to the persistence phenotype and how this might explain their drug-specific behavior. Furthermore, given the known involvement of these proteins in modulating *A*. *baumannii* virulence, it is hypothesized that persisters may exhibit higher virulence in vivo. To test this, persister and regular cells were injected in different quantities into *G*. *mellonella* larvae, and their survival rates were measured (Fig. 3). Analysis of the lethal dose for 50% (LD_50_) at each time point after infection showed a statistically significant difference between persister (2.75 × 10^5^ colony-forming unit (CFU)/larva) and regular cells (5.90 × 10^5^ CFU/larva) only at 24 h post-infection (Table 2). These data are in agreement with the common characteristic of persisters to revert to the regular phenotype upon cessation of the challenging stress. Therefore, the expression of virulence genes, such as *ompW* and *ompA*, is likely to return to baseline levels during infection, and, therefore, higher virulence would be expected to be transient.

**Table 2:** Comparison of LD_50_ values obtained by regular and persister (after meropenem exposure) *Acinetobacter baumannii* Acb-1 in *Galleria mellonella* larvae at different times of infection

Time of infection	*Acinetobacter baumannii* Acb-1	LD_50_ (CFU/larva)	95% CI (CFU/larva)	Relative mean potency estimates	Confidence interval for the relative mean potency estimates	Significance
24 h	Regular	5.90 × 10^5^	3.87 × 10^5^ – 9.41 × 10^5^	2.15	1.13–4.50	Yes
	Persister	2.75 × 10^5^	1.68 × 10^5^ – 4.48 × 10^5^	0.47	0.22–0.89	
48 h	Regular	2.32 × 10^5^	1.12 × 10^5^ – 5.00 × 10^5^	2.04	0.71–9.86	No
	Persister	1.10 × 10^5^	4.61 × 10^4^ – 2.71 × 10^5^	0.47	0.10–1.41	
72 h	Regular	2.19 × 10^5^	9.57 × 10^4^ – 5.30 × 10^5^	2.54	0.74–23.85	No
	Persister	8.63 × 10^4^	3.15 × 10^4^ – 2.52 × 10^5^	0.39	0.04–1.35	
96 h	Regular	2.19 × 10^5^	9.57 × 10^4^ – 5.30 × 10^5^	2.54	0.74–23.85	No
	Persister	8.63 × 10^4^	3.15 × 10^4^ – 2.52 × 10^5^	0.39	0.04–1.35	
120 h	Regular	2.19 × 10^5^	9.57 × 10^4^- 5.30 × 10^5^	2.54	0.74–23.85	No
	Persister	8.63 × 10^4^	3.15 × 10^4^ – 2.52 × 10^5^	0.39	0.04–1.35	

As previously mentioned, nutrients in the host environment are crucial for both host cells and bacterial pathogens. Iron, in particular, is an essential micronutrient. *Acinetobacter* spp. have evolved a high-affinity iron acquisition system through the use of acinetobactin to overcome iron sequestration. Studies have shown that mutants in genes involved in acinetobactin biosynthesis (*basD*) and transport (*bauA*) result in lower mortality rates in *G*. *mellonella* when compared to the wild type [42]. Therefore, it is plausible that OmpW, which is involved in acquiring iron [33], may play a role in the higher number of *G*. *mellonella* deaths by persister cells. This is especially noteworthy as *A. baumannii* has been shown to modulate OmpA levels in response to varying iron concentrations [25]. Furthermore, OmpW has been found to be important for adherence and invasion of mammalian host cells [24]. In this context, it must be noted that *A. baumannii* increases the secretion of OmpW and OmpA when challenged with sub-MIC tetracycline concentrations [43], and that OmpA is toxic to host cells when secreted [44].

Taking into account the increase in antimicrobial resistance, persistence, and evolution of virulence, the need for new drugs to treat *A*. *baumannii* infections is becoming increasingly important. Here we observed that the virulence genes *ompW* and *ompA* may additionally participate in the adaptation to drug exposure in persisters, and, therefore, considering the observed LD_50_ values obtained, exacerbate initial infection rates in vivo. Thus, OmpA and OmpW are promising candidates for further studies as potential drug targets not only for regular infective cells, but also for persisters of *A. baumannii*.

## Conclusions

Our study has revealed that both OmpA and OmpW play a role in the persistence phenotype of *A*. *baumannii*. Moreover, in vivo data indicate that persister cells are more virulent than the regular ones, as evidenced by their significantly lower LD_50_ value during early stages of infection. Further research is necessary to elucidate the mechanisms underlying the formation of persisters, which may then better support the development of drugs and/or immune-based strategies capable of impairing the essential functions of OmpA and OmpW.

## Materials and methods

### *Acinetobacter baumannii* strain

The experiments were carried out using the *A*. *baumannii* Acb-1 strain, which was previously isolated from tracheal aspirates by the Microbiology Sector of the Clinical Laboratory of Hospital São Lucas da PUCRS following the protocol approved by the Ethics Committee in Research (number 483,469). The Acb-1 strain was previously characterized as susceptible to meropenem, with a minimum inhibitory concentration (MIC) of 1 µg/mL and capable of forming persister cells at concentrations of up to 15 µg/mL [27]. The strain was stored at -80 °C in Brain Heart Infusion broth with 20% glycerol for further use.

### Persister cells assay

The persistence assays were performed in biological and experimental triplicate, according to the protocol described by Drescher et al. [45]. The Acb-1 strain was grown overnight in Lysogeny Broth (LB; 10 g/L tryptone, 5 g/L yeast extract, and 10 g/L NaCl), followed by a 1:50 dilution in 12 mL of LB and incubation at 37 °C until mid-exponential growth phase. Before the addition of the antimicrobial, aliquots of the culture were collected for RNA extraction and determination of the number of CFU/mL. The culture was diluted up to 10^− 6^, and 10 µL of each dilution was spotted on nutrient agar and incubated at 37 °C for 24 h. After removing the aliquots to determine the initial cell density and perform RNA purification, the remaining culture was exposed to meropenem (Sigma-Aldrich, Missouri, USA) at 15 µg/mL (15-fold MIC) at room temperature for 48 h. After 24 h and 48 h of exposure to meropenem, 1 mL aliquots were removed for RNA extraction and determination of persister fractions. After centrifugation (7 min at 9,660x*g*) of the aliquots and washing with 0.85% sterile saline solution to remove possible residues of antimicrobials, a serial decimal dilution was prepared and 10 µL of each dilution were spotted on nutrient agar. After 24 h of incubation at 37 °C, the number of CFU/mL of surviving cells was used to determine the persister fraction. A control culture was also grown without the addition of meropenem at the same conditions of time and temperature, to evaluate the number of CFU/mL at each time-point. The resistance phenotype was excluded upon confirmation of the MIC of the isolate (1 µg/mL) after persistence assays.

### RNA extraction

To extract RNA, the aliquots were centrifuged twice (5 min at 8,000x*g*), washed with 0.85% sterile saline solution, and then resuspended in 250 µL of sterile Milli-Q water. RNA extraction was performed with TRIzol LS reagent (Invitrogen, Massachusetts, USA) using a protocol adapted from the manufacturer. A 750 µL-aliquot of TRIzol was added, followed by an incubation period of 5 min at room temperature. Afterwards, 200 µL of chloroform was added and incubated for 10 min at room temperature, centrifuged (15 min at 12,000x*g* at 4 °C), and then 1 mL of 75% ethanol was added to the RNA pellet. The ethanol precipitated RNA was centrifuged (5 min at 7,500x*g* at 4 °C) and the pellet was air-dried for 5–10 min. The RNA was resuspended in 20 µL of RNase-free water (Invitrogen) and stored at − 80 °C. The concentration of total RNA was determined using NanoDrop™ One (Thermo Scientific, Massachusetts, USA). Finally, the RNA samples were treated with RNase-free DNase (Invitrogen).

### Relative quantitative real-time PCR (qPCR)

The relative expression levels of *adeB*, *ompA*, and *ompW* from *A. baumannii* were evaluated using 1 µg of purified total RNA as a template for reverse transcription, which was performed using the High-Capacity cDNA Reverse Transcription kit (Invitrogen). Amplification of the target genes was carried out using previously described oligonucleotide primers [46, 47]. To normalize the gene expression levels, the single copy housekeeping gene *rpoB* was used [47]. The qPCR analysis was performed in duplicate using the Platinum® SYBR® Green qPCR SuperMix-UDG kit (Invitrogen) in a StepOne thermocycler (Applied Biosystems, Massachusetts, USA).

### Virulence assay in *G. mellonella* larvae model

The in vivo virulence of *A. baumannii* regular and persister cells was assessed in *G*. *mellonella* larvae model according to Pinto et al. [48], with adaptations. The entire life cycle of *G. mellonella* was maintained in the laboratory at 28 °C and larvae were fed with laboratory diet, consisting of honey and several flours, until the day of experimentation. Groups of ten larvae in the final instar stage, weighing 220–260 mg, were used. The larvae were injected with 10 µL of bacterial suspension (10^2^ − 10^7^ CFU/larva) in PBS 1x pH 7.4 or with only PBS (vehicle control), using a 10 µL syringe (Hamilton Company, USA), into the hemocoel through the last right proleg. Subsequently, all larvae were incubated at 37 °C for 120 h in sterile Petri dishes. Every 24 h post-infection larvae survival was analyzed according to the response to touch stimuli. Experiments were performed in triplicate.

### Statistical analysis

The data obtained were statistically analyzed using the GraphPad Prism 9.0 software. Descriptive statistics of the data were performed, as well as the analysis of variance (two-way ANOVA) for independent samples, after which the Tukey test was performed to confirm the differences in each variable. A *p-value* < 0.05 was considered statistically significant for all tests.

In vivo experiments were analyzed through Kaplan-Meier survival curves obtained using GraphPad Prism 9.0. The LD_50_ value and respective 95% confidence intervals (95% CI) were calculated for regular and persister cells of *A. baumannii* using SPSS v.25 (SPSS Inc., Chicago, IL, USA) by fitting a logit regression to the data and reading the estimated cell number at a probability of 0.5. The difference between them was identified by relative median potency estimates. If the 95% confidence interval for the relative mean potency estimate does not include 1 on the untransformed scale, then the LD_50_ could be considered to be significantly different.

## Data Availability

The datasets used and/or analyzed during the current study are available from the corresponding author on reasonable request.

## References

[1] Leal MA, de Freitas-Vilela AA. Costs of healthcare-associated infections in an Intensive Care Unit. Rev Bras Enferm. 2021;74. 10.1590/0034-7167-2020-0275 33787794

[2] CDC. Centers for Disease Control and Prevention, Healthcare-associated Infections. Diseases and Organisms: Acinetobacter in Healthcare Settings. 2018. https://www.cdc.gov/hai/organisms/acinetobacter.html. Accessed 18 April 2022.

[3] ECDC. European Centre for Disease Prevention and Control, Healthcare-associated infections acquired in intensive care units: Annual Epidemiological Report for 2017. 2019. Surveillance Report. https://www.ecdc.europa.eu/sites/default/files/documents/AER_for_2017-HAI.pdf. Accessed 18 April 2022.

[4] Quartin AA, Scerpella EG, Puttagunta S, Kett DH (2013). A comparison of microbiology and demographics among patients with healthcare-associated, hospital-acquired, and ventilator-associated pneumonia: a retrospective analysis of 1184 patients from a large, international study. BMC Infect Dis.

[5] Barth VC, Rodrigues BA, Bonatto GD, Gallo SW, Pagnussatti VE, Ferreira CAS (2013). Heterogeneous persister cells formation in *Acinetobacter baumannii*. PLoS ONE.

[6] Lewis K (2010). Persister cells. Annu Rev Microbiol.

[7] Fauvart M, de Groote VN, Michiels J (2011). Role of persister cells in chronic infections: clinical relevance and perspectives on anti-persister therapies. J Med Microbiol.

[8] van den Bergh B, Fauvart M, Michiels J (2017). Formation, physiology, ecology, evolution and clinical importance of bacterial persisters. FEMS Microbiol Rev.

[9] Wilmaerts D, Windels EM, Verstraeten N, Michiels J (2019). General mechanisms leading to persister formation and awakening. Trends Genet.

[10] Salehi B, Ghalavand Z, Yadegar A, Eslami G (2021). Characteristics and diversity of mutations in regulatory genes of resistance-nodulation-cell division efflux pumps in association with drug-resistant clinical isolates of *Acinetobacter baumannii*. Antimicrob Resist Infect Control.

[11] Kashyap S, Kaur S, Sharma P, Capalash N (2021). Combination of colistin and tobramycin inhibits persistence of *Acinetobacter baumannii* by membrane hyperpolarization and down-regulation of efflux pumps. Microbes Infect.

[12] Sugawara E, Nikaido H (2012). OmpA is the principal nonspecific slow porin of *Acinetobacter baumannii*. J Bacteriol.

[13] Na S-H, Jeon H, Oh M-H, Kim Y-J, Chu M, Lee I-Y (2021). Therapeutic effects of inhibitor of *ompA* expression against carbapenem-resistant *Acinetobacter baumannii* strains. Int J Mol Sci.

[14] Viale AM, Evans BA. Microevolution in the major outer membrane protein OmpA of *Acinetobacter baumannii*. Microb Genom. 2020;6, e000381. 10.1099/mgen.0.000381 PMC7371106 32496178

[15] Sánchez-Encinales V, Álvarez-Marín R, Pachón-Ibáñez ME, Fernández-Cuenca F, Pascual A, Garnacho-Montero J (2017). Overproduction of outer membrane protein A (OmpA) by *Acinetobacter baumannii* is a risk factor for nosocomial pneumonia, bacteremia and mortality increase. J Infect Dis.

[16] Hong H, Patel DR, Tamm LK, van den Berg B (2006). The outer membrane protein OmpW forms an eight-stranded β-barrel with a hydrophobic channel. J Biol Chem.

[17] Brambilla L, Morán-Barrio J, Viale AM (2014). Expression of the *Escherichia coli ompW* colicin S4 receptor gene is regulated by temperature and modulated by the H-NS and StpA nucleoid-associated proteins. FEMS Microbiol Lett.

[18] Wu L, Lin X, Peng X (2009). From proteome to genome for functional characterization of pH-dependent outer membrane proteins in *Escherichia coli*. J Proteome Res.

[19] Huang W, Wang S, Yao Y, Xia Y, Yang X, Long Q (2015). OmpW is a potential target for eliciting protective immunity against *Acinetobacter baumannii* infections. Vaccine.

[20] Lei L, Yang F, Zou J, Jing H, Zhang J, Xu W (2019). DNA vaccine encoding OmpA and pal from *Acinetobacter baumannii* efficiently protects mice against pulmonary infection. Mol Biol Rep.

[21] Mehdinejadiani K, Hashemi A, Bandehpour M, Rahmani H, Ranjbar MM, Yardel V (2021). Evaluation of the new outer membrane protein A epitope-based vaccines for mice model of *Acinetobacter baumannii* associated pneumonia and sepsis infection. Iran J Allergy Asthma Immunol.

[22] Tao Y, Duma L, Rossez Y (2021). Galleria mellonella as a good model to study Acinetobacter baumannii pathogenesis. Pathogens.

[23] Liu D, Liu Z-S, Hu P, Cai L, Fu B-Q, Li Y-S (2016). Characterization of surface antigen protein 1 (SurA1) from *Acinetobacter baumannii* and its role in virulence and fitness. Vet Microbiol.

[24] Gil-Marqués ML, Pachón J, Smani Y (2022). iTRAQ-based quantitative proteomic analysis of *Acinetobacter baumannii* under hypoxia and normoxia reveals the role of OmpW as a virulence factor. Microbiol Spectr.

[25] Liu H, Cao CY, Qiu FL, Huang HN, Xie H, Dong R (2021). Iron-rich conditions induce OmpA and virulence changes of *Acinetobacter baumannii*. Front Microbiol.

[26] Bigger JW (1944). Treatment of staphylococcal infections with penicillin by intermittent sterilisation. The Lancet.

[27] Gallo SW, Ferreira CAS, de Oliveira SD (2017). Combination of polymyxin B and meropenem eradicates persister cells from *Acinetobacter baumannii* strains in exponential growth. J Med Microbiol.

[28] Choi CH, Lee JS, Lee YC, Park TI, Lee JC (2008). Acinetobacter baumannii invades epithelial cells and outer membrane protein A mediates interactions with epithelial cells. BMC Microbiol.

[29] Gaddy JA, Tomaras AP, Actis LA (2009). The *Acinetobacter baumannii* 19606 OmpA protein plays a role in biofilm formation on abiotic surfaces and in the interaction of this pathogen with eukaryotic cells. Infect Immun.

[30] Nie D, Hu Y, Chen Z, Li M, Hou Z, Luo X (2020). Outer membrane protein A (OmpA) as a potential therapeutic target for *Acinetobacter baumannii* infection. J Biomed Sci.

[31] Wu X, Chavez JD, Schweppe DK, Zheng C, Weisbrod CR, Eng JK (2016). In vivo protein interaction network analysis reveals porin-localized antibiotic inactivation in *Acinetobacter baumannii* strain AB5075. Nat Commun.

[32] Shahryari S, Talaee M, Haghbeen K, Adrian L, Vali H, Zahiri SH (2021). New provisional function of OmpA from *Acinetobacter* sp. strain SA01 based on environmental challenges. mSystems.

[33] Catel-Ferreira M, Marti S, Guillon L, Jara L, Coadou G, Molle V (2016). The outer membrane porin OmpW of *Acinetobacter baumannii* is involved in iron uptake and colistin binding. FEBS Lett.

[34] López-Rojas R, Smani Y, Pachón J (2013). Treating multidrug-resistant *Acinetobacter baumannii* infection by blocking its virulence factors. Expert Rev Anti Infect Ther.

[35] Lin X-M, Yang J-N, Peng X-X, Li H (2010). A novel negative regulation mechanism of bacterial outer membrane proteins in response to antibiotic resistance. J Proteome Res.

[36] Kashyap S, Sharma P, Capalash N (2022). Tobramycin stress induced differential gene expression in *Acinetobacter baumannii*. Curr Microbiol.

[37] Nandi B, Nandy RK, Sarkar A, Ghose AC (2005). Structural features, properties and regulation of the outer-membrane protein W (OmpW) of *Vibrio cholerae*. Microbiol.

[38] Shahryari S, Mohammadnejad P, Noghabi KA (2021). Screening of anti- *Acinetobacter baumannii* phytochemicals, based on the potential inhibitory effect on OmpA and OmpW functions. R Soc Open Sci.

[39] Soojhawon I, Pattabiraman N, Tsang A, Roth AL, Kang E, Noble SM (2017). Discovery of novel inhibitors of multidrug-resistant *Acinetobacter baumannii*. Bioorg Med Chem.

[40] Pu Y, Zhao Z, Li Y, Zou J, Ma Q, Zhao Y (2016). Enhanced efflux activity facilitates drug tolerance in dormant bacterial cells. Mol Cell.

[41] Huang L, Sun L, Xu G, Xia T (2008). Differential susceptibility to carbapenems due to the AdeABC efflux pump among nosocomial outbreak isolates of *Acinetobacter baumannii* in a chinese hospital. Diagn Microbiol Infect Dis.

[42] Gaddy JA, Arivett BA, McConnell MJ, López-Rojas R, Pachón J, Actis LA (2012). Role of acinetobactin-mediated iron acquisition functions in the interaction of *Acinetobacter baumannii* strain ATCC 19606 with human lung epithelial cells, *Galleria mellonella* caterpillars, and mice. Infect Immun.

[43] Yun S-H, Choi C-W, Park S-H, Lee JC, Leem S-H, Choi J-S (2008). Proteomic analysis of outer membrane proteins from *Acinetobacter baumannii* DU202 in tetracycline stress condition. J Microbiol.

[44] Uppalapati SR, Sett A, Pathania R (2020). The outer membrane proteins OmpA, CarO, and OprD of *Acinetobacter baumannii* confer a two-pronged defense in facilitating its success as a potent human pathogen. Front Microbiol.

[45] Drescher SPM, Gallo SW, Ferreira PMA, Ferreira CAS, Oliveira SD (2019). *Salmonella enterica* persister cells form unstable small colony variants after in vitro exposure to ciprofloxacin. Sci Rep.

[46] Dou Y, Song F, Guo F, Zhou Z, Zhu C, Xiang J (2017). *Acinetobacter baumannii* quorum-sensing signalling molecule induces the expression of drug-resistance genes. Mol Med Rep.

[47] Rumbo C, Gato E, López M, Ruiz de Alegría C, Fernández-Cuenca F, Martínez-Martínez L (2013). Contribution of efflux pumps, porins, and β-lactamases to multidrug resistance in clinical isolates of *Acinetobacter baumannii*. Antimicrob Agents Chemother.

[48] Pinto HB, Brust FR, Macedo AJ, Trentin DS (2020). The antivirulence compound myricetin possesses remarkable synergistic effect with antibacterials upon multidrug resistant *Staphylococcus aureus*. Microb Pathog.

